# Crowded

**Published:** 1874-01

**Authors:** 


*Crowded.*

No, not much! Only one doctor to every six hundred inhabitants, "good bad and indifferent," in the United States!! That's a small affair, to some; especially if *they* should feel it a part of "*destiny*" to *cheapen* medical education and run the profession in the next decade, to "smash." There's "plenty of room at the top" boys, but take care if you surge and squirm "with the million" at the bottom, with quacks, deceivers, trimmers, tricksters and hosts of irregulars, pushing, pulling and gouging the life out of you in the struggle, to get one good, wholesome, independent breath beyond the confines of a merciless impecuniosity.

Two regiments a year! Call the roll, for one more decade will end the struggle. "The begining of the end" of all prosperity and honor to the profession has come—unless, unless the medical tub-mills now grinding surely and constantly break down! W. T. G.

